# Ultrasonographic and Electromyographic Evaluation of the Effects of Deep Versus Superficial Dry Needling on Myofascial Trigger Points in Patients with Neck Pain: A Triple-Blind Randomized Clinical Trial

**DOI:** 10.3390/bios16100583

**Published:** 2026-10-18

**Authors:** Anas Mohammed Alhakami, Diyaa Abdul Rauf Algazwi, Majdi Habib Al-Awjami, Walaa Hamdy Elsayed, Ali M. Alshami

**Affiliations:** 1 Department of Physical Therapy, College of Applied Medical Sciences, Imam Abdulrahman Bin Faisal University, Dammam 31441, Saudi Arabia; whelsayed@iau.edu.sa (W.H.E.); alshami@iau.edu.sa (A.M.A.); 2 Department of Medical Imaging, Qatif Health Network, Qatif 32654, Saudi Arabia; dmohammed-algazwi@moh.gov.sa; 3 Department of Medical Imaging, Prince Mohammed Bin Fahad Hospital of Blood Disorder, Qatif 32643, Saudi Arabia; malawjami@moh.gov.sa

**Keywords:** dry needling, myofascial trigger points, neck pain, ultrasonography, surface electromyography, randomized clinical trial

## Abstract

**Background**: The physiological differences between deep dry needling (DDN) and superficial dry needling (SDN) for myofascial trigger points (MTrPs) remain unclear. This randomized clinical trial compared the impact of DDN and SDN on ultrasonographic, surface electromyographic (sEMG), and clinical outcomes in patients with non-specific neck pain and upper trapezius MTrPs. **Methods**: Forty-four participants were randomly allocated to DDN (50 mm) or SDN (13 mm), with 22 participants in each group. Participants received four treatment sessions over 2 weeks. Ultrasonographic outcomes included upper trapezius stiffness, thickness, and blood-flow parameters. sEMG outcomes included maximum voluntary isometric contraction (MVIC), root mean square (RMS), and median frequency (MDF). Pain intensity was measured using the Visual Analogue Scale (VAS) and disability using the Neck Disability Index (NDI). Outcomes were assessed at baseline, following 2 weeks of intervention, and during the 6-week follow-up. **Results**: No statistically significant between-group differences were detected for the ultrasonographic outcomes. For sEMG, the time-specific contrast for active RMS at the 6-week follow-up was nominally significant (DDN minus SDN: 1.09, 95% CI 0.06–2.13; *p* = 0.039), whereas the remaining between-group sEMG comparisons were not statistically significant. A small within-group reduction in muscle stiffness was observed in the SDN group at 2 weeks (−0.09, 95% CI −0.17 to −0.001). MVIC increased in both groups at 6 weeks, and active RMS decreased within the SDN group. Both groups showed reductions in pain and disability; adjusted VAS changes ranged from −3.04 to −3.35 cm and adjusted NDI changes ranged from −4.5 to −6.5 points across groups and follow-up assessments. The isolated *p* = 0.039 finding is interpreted cautiously because multiple outcomes were examined without formal multiplicity adjustment. **Conclusions**: Both DDN and SDN were associated with improvements in pain and disability, with no statistically significant between-group differences detected for these clinical outcomes in this sample. Most ultrasonographic and sEMG between-group comparisons were also non-significant, although a nominal difference in active RMS was observed at 6 weeks. Because this trial was not designed as an equivalence or non-inferiority study, the findings should not be interpreted as establishing therapeutic equivalence. The absence of consistent ultrasonographic changes suggests that mechanisms beyond measurable local structural or vascular changes may contribute to the observed clinical improvements; such mechanisms were not directly measured in this study.

## 1. Introduction

**Figure 1:**
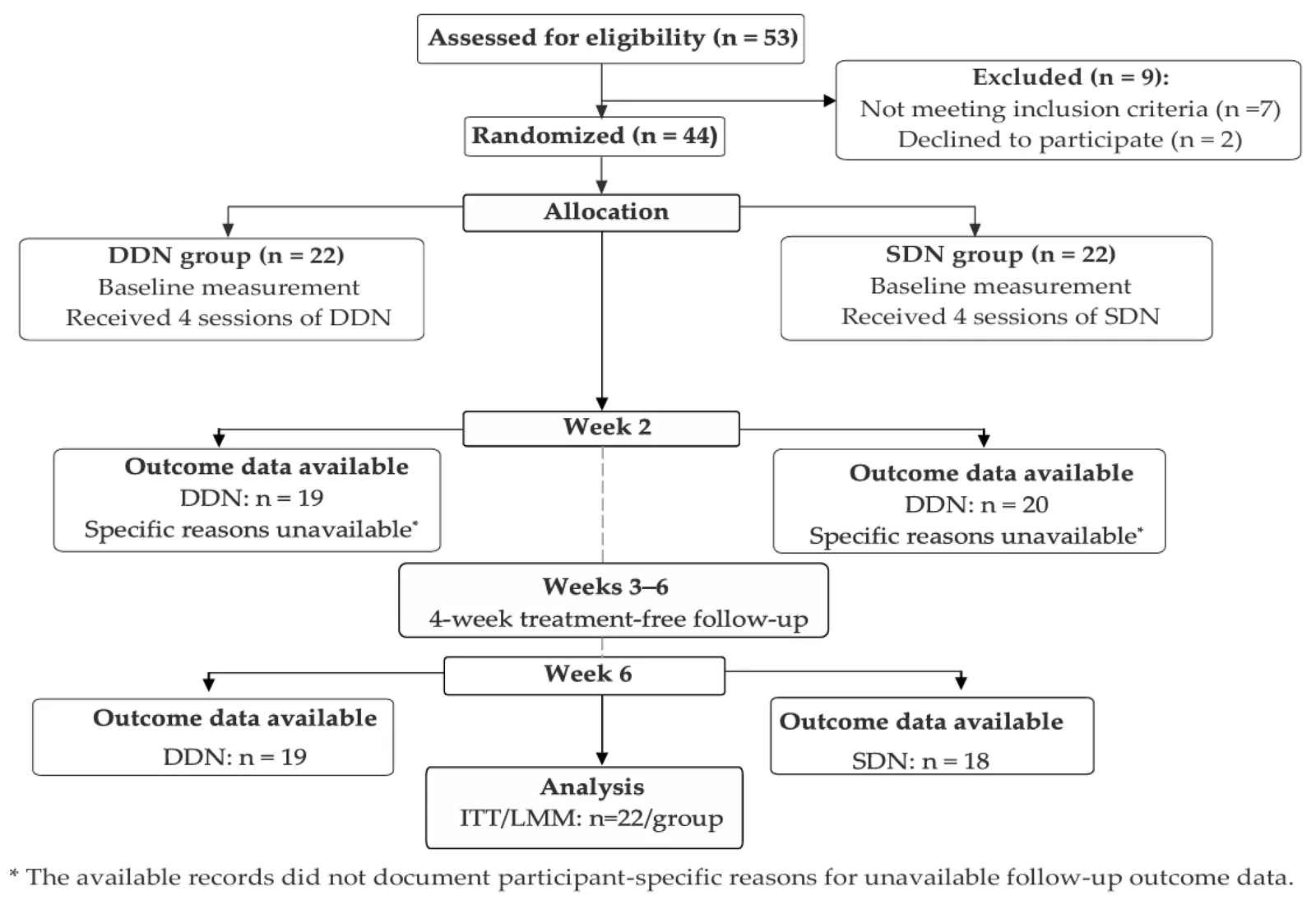
CONSORT flow diagram of participant recruitment, allocation, follow-up, and analysis.

Myofascial pain syndrome (MPS) is a frequently encountered musculoskeletal condition, with reported lifetime prevalence estimates varying between 30% and 85% contingent upon the demographic group studied figure [1,2]. Approximately 69% of individuals in Saudi Arabia indicate that they have experienced symptoms of MPS at some point in their lives [3]. The widely recognized definition of MPS was developed by Simons and Travell in 1983 as localized soreness linked to the existence of one or many myofascial trigger points (MTrPs) [4]. MTrPs are defined by sensitive nodules located inside tense bands of skeletal muscle, frequently resulting in regional tenderness, referred pain patterns, involuntary local twitch responses, and restrictions in joints’ range of motion [5]. In patients with neck pain, the upper trapezius muscle exhibits the greatest prevalence of MTrPs, impacting 93.75% of cases [6].

The mechanical and physiological characteristics of MTrPs have been examined through diverse modalities in relation to the Simons–Travell energy-crisis hypothesis. This model proposes that sustained muscle contraction may impair regional blood flow, producing local ischemia, metabolic stress, and excessive acetylcholine release at the neuromuscular junction [4,7]. However, MTrP pain is not considered to be explained exclusively by a local energy-crisis mechanism; peripheral nociceptive sensitization, altered motor-endplate activity, central pain-processing mechanisms, and broader biopsychosocial influences have also been proposed [8]. Thus, the Simons–Travell model remains influential but is not the only framework for interpreting MTrP persistence and treatment response.

Physiotherapy management of MTrPs includes non-invasive approaches such as manual therapy and exercise [9,10] and invasive approaches such as dry needling (DN) [9,11]. DN may be applied superficially or deeply according to the intended penetration depth [12,13]. Prior comparative trials of DDN and SDN have reported mixed findings, with broadly similar improvements in pain and disability in some studies and outcome- or follow-up-specific differences in others [14,15,16,17,18]. Earlier systematic-review evidence also noted heterogeneity in comparative findings [15], while a recent systematic review of eight randomized trials reported short-term improvements with both techniques without consistently demonstrated clinically meaningful differences between them [19]. A systematic review and meta-analysis of dry needling for chronic neck pain likewise reported improvements in pain and function while emphasizing heterogeneity across protocols and comparators [20]. Objective longitudinal evidence concerning local muscle architecture, vascular characteristics, and sEMG responses remains limited. This gap provided the rationale for the present study, which compared these two DN techniques using ultrasonographic, sEMG, pain, and disability outcomes.

It was hypothesized that DDN would produce greater improvements in the properties of skeletal muscles than SDN because the deeper needle insertion enables direct access to the intramuscular trigger point [21]. The direct targeting of MTrPs was expected to induce greater improvement in muscle thickness, stiffness, and blood flow [22].

## 2. Materials and Methods

### 2.1. Study Design, Setting, and Ethical Considerations

This triple blind two-arm, parallel-group randomized clinical trial was conducted at Qatif Central Hospital, Eastern Province, Saudi Arabia. The assessors and participants were unaware of the group assignment, and the therapist was unaware of the outcome measures. Operationally, participants and outcome assessors were blinded to group allocation, whereas the treating therapist knew which intervention was delivered but remained blinded to outcome measurements. The study was registered with ClinicalTrials.gov (NCT07637552) and approved by the Ethics Committee of Imam Abdulrahman Bin Faisal University (IRB-PGS-2024-03-872) and Qatif Central Hospital (QCH-SRECO 50/2024). The available study records did not permit independent verification of the registry submission date relative to first participant enrollment; therefore, the manuscript does not rely on the term “prospectively registered.” The study followed CONSORT guidance and the Declaration of Helsinki, and all participants provided written informed consent. The dry needles used in this study received Saudi Food and Drug Authority approval (MDMA-2-2024-3319).

### 2.2. Sample Size Calculation

Sample size calculation was conducted a priori using G*Power version 3.1.9.6 (University of Dusseldorf, Dusseldorf, Germany). A repeated-measures analysis of variance for the within-between interaction between two groups and three measurement timepoints was assumed, with effect size f = 0.25, α = 0.05, power (1 − β) = 0.90, correlation among repeated measurements r = 0.50, and nonsphericity correction ε = 1.00. The minimum analyzable sample was 36 participants (18 per group). The original recruitment target was obtained by adding 20% to this minimum (36 × 1.20 = 43.2) and rounding up to 44 participants (22 per group). This reports the calculation used in the study; conventional inflation by division by 0.80 would yield 45 participants.

### 2.3. Participants

Patients were diagnosed with non-specific neck pain at an orthopedic clinic or primary health care center and referred to the Department of Physiotherapy. Males or females aged 18–65 years were considered eligible for the study if they reported acute or chronic neck pain and had at least one active MTrP in the upper trapezius muscle. In the case of bilateral active MTrPs, the more sensitive side as assessed by palpation was selected for examination and therapy. Participants were excluded if they had a prior whiplash injury, cervical spine surgery, cervical radiculopathy or fibromyalgia. Other reasons for exclusion included having received pain-related treatment (physiotherapy or medication) in the week prior to enrollment, having a diagnosed psychiatric condition (including depression or anxiety), or having a contraindication to DN (e.g., needle phobia or anticoagulant medication). The available study documentation does not record a protocol-mandated diagnostic imaging examination or other standardized additional diagnostic work-up performed specifically for study eligibility. This does not exclude clinically indicated investigations before referral; such investigations were not systematically documented as study procedures.

The active MTrPs were detected by the treating therapist (AMA) based on the following criteria developed by Simons et al. [23] : a hypersensitive tender spot within a palpable taut band, local or referred pain upon compression of the spot that the patient recognized as familiar, and/or restricted neck range of motion [23]. Inter- and intra-rater reliability for MTrP identification has been reported as moderate to high (ICC = 0.62–0.81) [24]. These diagnostic criteria are supported by international expert consensus from a Delphi study [5].

### 2.4. Randomization and Concealment

Participants were randomly assigned on a 1:1 basis to 2 parallel intervention groups, the DDN group and the SDN group. To maintain independence of the allocation procedure, a researcher not engaged in the intervention created 44 distinct random numbers using an online randomization tool from a website. The numbers produced were listed one by one on cards, which were folded and placed in sealed, opaque envelopes. Each participant chose one envelope, and the envelope number was used for assignment to the DDN or SDN group.

### 2.5. Interventions

The treating therapist (AMA), who is certified in DN with 13 years of experience, performed the intervention. Each group received four sessions of DDN or SDN over 2 weeks (two sessions per week), with a minimum of 48 h between sessions [25]. After completion of treatment in Week 2, participants entered a 4-week treatment-free follow-up period, with the final assessment performed at Week 6.

DDN group. Patients lay supine while the therapist palpated the upper trapezius on both sides to locate active MTrPs. When more than one active MTrP was found, the therapist treated the one the patient reported as causing the most severe symptoms. After the skin was cleaned with an alcohol swab, a sterile, dry needle with a diameter of 0.25 mm and a length of 50 mm (Wujiang City Cloud & Dragon Medical Device Company, Suzhou, China) was inserted deep into the MTrP and moved up and down 3 to 5 times using the fast-in/fast-out technique [26] .

SDN group. The same patient positioning and MTrP identification used in the DDN group were applied. A single-use sterile needle (0.25 mm diameter, 13 mm length; Wujiang City Cloud & Dragon Medical Device Company, China) was inserted minimally over the identified MTrP and moved vertically 3–5 times using the fast-in/fast-out technique [26]. The protocol intended the needle to remain superficial without deliberate penetration of the underlying muscle or elicitation of a local twitch response. Actual insertion depth was not instrumentally measured; therefore, needle length should not be interpreted as insertion depth. Both groups received the same prescribed number of needle movements (3–5 repetitions). Exact manoeuvre duration in seconds was not recorded, so identical total stimulation duration cannot be confirmed beyond the standardized repetition range.

### 2.6. Outcome Measures

The outcome measures were assessed at three prespecified timepoints: baseline, immediately after completion of the 2-week treatment period (Week 2), and at Week 6, corresponding to 4 weeks after treatment completion. Ultrasonographic assessments were performed by a physician with more than 5 years of experience in musculoskeletal ultrasonography. The sEMG and other outcomes were evaluated by physiotherapists with more than 20 years of professional experience. Only the affected side was analyzed. Adverse events were recorded immediately after each treatment session and monitored for up to 27 h post-intervention. Events were considered minor when they were transient, self-limiting, and did not require additional medical treatment or withdrawal from the study.

#### 2.6.1. Primary Outcomes

##### Ultrasonography

Patients were asked to sit in a relaxed upright position with both forearms pronated on the armrests. An ultrasound machine (Model LOGIQ E9 R6, GE Healthcare Technologies Inc., Chicago, IL, USA) was used to examine the elasticity, thickness, and blood flow of the upper trapezius muscle.

Strain elastography was used to measure the stiffness of the upper trapezius muscle. Images were obtained with a linear transducer positioned longitudinally along the muscle fibers, while applying gentle, rhythmic compression to ensure adequate tissue deformation. Care was devoted to ensuring uniform pressure and proper probe alignment during the assessment. Regions of interest (ROIs) were identified inside the upper trapezius muscle, measuring 5 mm^2^. The designated ROI was situated in the subcutaneous tissue, whilst the secondary ROI was located in the upper trapezius muscle fibers. The strain ratio was assessed [27]. In this imaging configuration, softer tissues are depicted in red, whereas stiffer tissues are illustrated in blue. Strain elastography demonstrated moderate to high reliability (ICC = 0.70–0.81) [28].

The study documentation specifies standardized probe orientation, compression technique, and ROI placement; however, it does not record a prespecified number of repeated elastography acquisitions or a formal averaging procedure. This detail is therefore reported as unavailable rather than inferred retrospectively.

B-mode ultrasonography was employed to assess the thickness of the upper trapezius at rest, with the probe oriented parallel to the muscle fibers. Thickness was defined as the maximum distance (mm) between the superficial and deep edges of the muscle. The MTrP was identified as a focal hypoechoic (darker) area with a heterogeneous echotexture. B-mode sonography is highly reliable for assessing muscle thickness (ICC = 0.98) [29].

Color Doppler ultrasonography was used to assess blood flow within and around the upper trapezius MTrPs during systole and diastole. The sonographer applied two levels of transducer pressure to the muscle: minimal pressure, with the transducer barely touching the skin, and moderate pressure, producing visible compression of the muscle. The peak systolic velocity (PSV; cm/s), end diastolic velocity (EDV; cm/s), and resistance index (RI) were calculated. The reliability of Doppler ultrasonography in measuring blood flow is moderate to good (ICC = 0.67–0.87) [29].

##### Surface Electromyography

Muscle activity of the afflicted upper trapezius was recorded with a Delsys Trigno Avanti wireless surface electromyography (sEMG) system (Delsys Inc., Natick, MA, USA). Skin preparation and sensor placement were done according to guidelines of Surface ElectroMyoGraphy for the Non-Invasive Assessment of Muscles (SENIAM), modified for integrated wireless EMG sensors [30].

One sEMG sensor was placed on the upper trapezius, about at the midway of the line from the acromion to the C7 spinous process, with the longitudinal axis parallel to the direction of the muscle fibers. The location of recording was shaved, lightly abraded and cleaned with an alcohol swab, as necessary, and the sensor was secured with adhesive tape to reduce skin impedance.

The signals were recorded with a sample frequency of 1000 Hz and bandwidth of 20–450 Hz using the EMGworks Acquisition program (Version 4.8.0). EMG works Analysis software (Version 1.0.0.0) was used for further signal processing. From this, the root mean square (RMS) amplitude and median frequency (MDF) were calculated.

sEMG recordings were performed in a consistent way in three conditions: rest, activity and resistance. The muscle activity was measured for 5 s while the subjects sat comfortably on a chair with supported arms during the resting state. During the exercise condition, the arms were positioned at 30° of abduction and participants executed an unloaded shoulder shrug, maintaining the position for 5 s. Resistance was assessed by having the subjects complete a maximal voluntary isometric contraction (MVIC) by shrugging their shoulders against standardized manual resistance with both arms abducted at 30°. Contraction duration was 5 s. Each condition was performed thrice with a minute rest period between subsequent repetitions. For statistical analysis, the average of the three trials for each condition was utilized.

Skin preparation was undertaken to reduce electrode–skin impedance; however, the study records do not document a numerical impedance threshold or post-preparation impedance measurement. RMS and MDF values were derived from the recorded sEMG signals for the specified tasks and were not normalized to each participant’s MVIC; MVIC was analyzed as a separate normalized outcome.

#### 2.6.2. Secondary Outcomes

##### Pain Intensity

The current pain severity was evaluated using a visual analog scale (VAS) comprising a 10-cm horizontal line. A score of 0 indicates “absence of pain,” while a score of 10 indicates “the utmost degree of pain.” The VAS demonstrates excellent reliability (ICC = 0.97) and valid instrument for evaluating musculoskeletal pain [31].

##### Functional Disability

The Neck Disability Index (NDI) was employed to assess the influence of neck pain on an individual’s everyday functions. The instrument comprises 10 items and is divided into 10 sections. Each item is assigned up to 5 points, with 0 points corresponding to “no limitation” and 5 points corresponding to “complete limitation.” The NDI is scored out of a maximum of 50 points, with higher scores indicating greater neck-related disability. Scores of 0–4 reflect no disability, 5–14 mild disability, 15–24 moderate disability, 25–34 severe disability, and scores above 35 complete disabilities [32]. The NDI is valid and has good reliability (ICC = 0.88) for measuring neck-related functional disability [33].

### 2.7. Statistical Analysis

Statistical analysis was performed using the Statistical Package for the Social Sciences (SPSS) version 31 (IBM Corporation, Armonk, NY, USA) following the intention-to-treat (ITT) principle. Descriptive statistics were computed for all outcome measures. Continuous data are reported as mean ± standard deviation (SD) for each treatment group at each assessment timepoint. Baseline comparability by distribution and kind of variable was assessed between groups. Continuous variables with normal distribution were studied using independent-samples *t*-tests while continuous variables not meeting the assumption of normality were analyzed with Mann–Whitney U test. Categorical variables were compared using the χ2 test or Fisher’s exact test as applicable. The distributional assumption of the model residuals was tested using the Shapiro–Wilk test and eye inspection of the Q–Q plots.

Model residual distributions were assessed using the Shapiro–Wilk test and Q–Q plots. Although transformations were explored during model checking, the final SPSS output used for the reported active RMS contrasts analyzes active RMS on the original change-from-baseline scale (Act_RMS_Diff). Accordingly, the active RMS estimates and 95% CIs reported in the original RMS unit (μV).

A linear mixed model (LMM) with restricted maximum likelihood estimation was used to assess treatment effects across time. A separate model was developed for each outcome. Change from baseline was the dependent variable, and the matching baseline value was included as a covariate. The LMM used all available observations under the Missing at Random (MAR) assumption without single-value imputation or casewise deletion [34,35]. Fixed effects included assessment time (Week 2 post-treatment and Week 6 follow-up), treatment group (DDN versus SDN), and the time-by-group interaction. Estimated marginal means with 95% confidence intervals (CIs) were obtained for each group and timepoint. Statistical significance was set at *p* < 0.05 (two-tailed). No formal multiplicity adjustment was prespecified across the multiple ultrasonographic and sEMG endpoints; therefore, isolated *p* values near 0.05 were interpreted cautiously as exploratory rather than confirmatory evidence. Adverse-event incidence was compared using the chi-square test or Fisher’s exact test, as appropriate.

## 3. Results

### 3.1. Participants Recruitment and Flow

Fifty-three patients were screened for this study. A total of 44 patients satisfied the criteria and were recruited (Figure 1).

The baseline demographic characteristics of the patients are presented in Table 1. No statistically significant baseline differences were detected for the reported demographic variables. Mean neck-pain duration was 39.52 months in the DDN group and 56.20 months in the SDN group; these values are group means rather than an observed range. Physical-activity information was unavailable for two DDN participants; this is clarified in the Table 1 footnote.

### 3.2. Outcome Measures Findings

Table 2 presents the mean ± SD values of all outcome measures at baseline, Week 2 post-treatment, and Week 6 follow-up. The randomized sample remained DDN n = 22 and SDN n = 22. Baseline observations were available for all participants; non-missing observed outcome measurements were available for DDN n = 19 and SDN n = 20 at Week 2, and DDN n = 19 and SDN n = 18 at Week 6. These are observed-data counts rather than revised group sample sizes. All randomized participants were retained in the LMM/ITT framework using available repeated observations under the MAR assumption.

#### 3.2.1. Primary Outcomes Findings

The LMM results for the primary outcomes are presented in Table 3. Omnibus group-by-time interactions were not statistically significant for the ultrasonographic parameters or for MVIC, rest RMS, rest MDF, or active MDF. The time-specific contrast for active RMS at Week 6 was nominally significant (DDN minus SDN: 1.09, 95% CI 0.06–2.13; *p* = 0.039), whereas the Week 2 contrast was not significant. Within groups, active RMS decreased in the SDN group at Week 2 and Week 6, MVIC increased at Week 6 in both groups, and a small reduction in muscle stiffness was observed in the SDN group at Week 2 (−0.09, 95% CI −0.17 to −0.001). Because multiple physiological outcomes were examined without formal multiplicity adjustment, the isolated active-RMS *p* = 0.039 is interpreted cautiously as exploratory. The numerical entries in Table 3 have been retained to preserve the originally reported statistical table, while the narrative has been revised to avoid interpretations that conflict with the reported estimates and confidence intervals.

#### 3.2.2. Secondary Outcomes Findings

The LMM results for the secondary outcomes are presented in Table 4. The group-by-time interaction was not statistically significant for VAS (F = 0.26, *p* = 0.77) or NDI (F = 0.596, *p* = 0.556), and no statistically significant between-group difference was detected at Week 2 or Week 6. Adjusted VAS changes ranged from −3.04 to −3.35 cm and adjusted NDI changes ranged from −4.5 to −6.5 points across groups and follow-up assessments. Exact estimates are reported rather than applying a single threshold to all timepoints.

For complementary visualization of these clinical outcomes, mean VAS and NDI scores for the DDN and SDN groups across baseline, Week 2, and Week 6 are presented in Supplementary Figures S1 and S2, respectively.

### 3.3. Adverse Events

Table 5 presents the incidence of recorded adverse events, which were predominantly minor and transient. DDN was associated with more bleeding (36%), bruising (18%), pain during needling (77%), and pain after needling (63%). In the SDN group, pain during needling was reported by 41% and pain after needling by 27% of participants. Percentages represent participants who experienced the specified event at least once across the four treatment sessions, rather than per-session event rates.

## 4. Discussion

To our knowledge, this randomized controlled study directly compared DDN and SDN using objective physiological outcomes, including ultrasonography and sEMG, together with patient-reported pain and disability. No statistically significant between-group differences were detected for the ultrasonographic outcomes or for pain and disability at Week 2 or Week 6. A nominally significant time-specific between-group difference was observed for active RMS at Week 6 (*p* = 0.039), alongside within-group decreases in active RMS in the SDN group. Because multiple outcomes were examined without multiplicity adjustment, this isolated finding should be interpreted cautiously. Both groups showed reductions in pain and disability; however, in the absence of a sham or untreated control group, the extent to which these within-group changes are attributable specifically to dry needling cannot be determined.

These findings corroborate those reported [36] in which a single session of DDN applied to the upper trapezius muscle did not result in significant changes in muscle thickness. Similarly, ref. [37] found no significant alterations in upper trapezius muscle stiffness following acupuncture interventions with MTrPs in patients with MPS. However, unlike the present study, the intervention was applied to traditional acupuncture points rather than directly targeting MTrPs. Conversely, ref. [38] reported a marked reduction in PSV following DN, while no changes were observed in EDV or RI, suggesting a selective vascular response. In contrast, ref. [36] exhibited significant immediate improvements in both vascular parameters (PSV, EDV, and RI) and muscle stiffness following DDN. Discrepancies between studies suggest that the effects of DN on muscle vascularity and mechanical properties may be time-dependent and influenced by the timing of the outcome assessment.

The absence of significant ultrasonographic changes in the current study may be explained by both physiological and methodological considerations. Although ultrasonography can identify MTrPs as small hypoechoic (darker) elliptical regions, accurately differentiating these localized abnormalities from the surrounding muscle tissue remains challenging, which may reduce the sensitivity of ultrasounds in detecting subtle treatment-induced changes [39]. Furthermore, the absence of ultrasonographic changes may reflect physiological adaptations to treatment that do not produce detectable alterations in muscle morphology or echogenicity on a conventional B-mode ultrasonography. Alternatively, it is possible that the intervention did not produce measurable structural changes within the muscle itself, despite improvements in clinical outcomes through mechanisms such as pain modulation.

Regarding sEMG outcomes, MVIC increased within both groups in Week 6. Active RMS decreased within the SDN group at Weeks 2 and 6, and the Week 6 time-specific contrast differed between groups (DDN minus SDN: 1.09, 95% CI 0.06–2.13; *p* = 0.039). This isolated finding may indicate a difference in muscle activation at that assessment, but it should be interpreted cautiously because numerous physiological outcomes were tested without multiplicity adjustment. The study therefore does not provide confirmatory evidence of a consistent between-group sEMG effect.

Both groups demonstrated reductions in pain (VAS) and disability (NDI), with no statistically significant between-group differences detected for these clinical outcomes. Adjusted VAS changes ranged from −3.04 to −3.35 cm across groups and timepoints, exceeding the cited pain MCID of 0.8 cm [40]. Adjusted NDI changes ranged from −4.5 to −6.5 points and overlap the cited MCID range of 3.5–5 points [41,42]. These within-group changes should not be interpreted as proving treatment-specific efficacy because the trial did not include sham or untreated control. Prior comparative studies have likewise reported improvements following both approaches [14,16,19].

The present study found that DDN was associated with a much higher incidence of bleeding (36.4%), pain during needling (77.3%), and pain after needling (63.6%) compared to SDN, while no significant differences were observed for other adverse events. Consistent with the current results, previous studies have identified bleeding, bruising, pain during needling, and post-needling soreness as the most frequently reported adverse events associated with DDN [43,44]. Significantly, all the adverse incidents documented herein can be categorized as minor or temporary, which are recognized to occur regularly in clinical settings [25,43]. Although these occurrences are generally modest and self-resolving, the reduced occurrence in the SDN group indicates that SDN could be a safer and more tolerable option, especially for individuals with low pain thresholds or those predisposed to bleeding.

### 4.1. Theoretical Implications: Challenging the Simons and Travell Integrated Hypothesis

The present findings should not be interpreted as demonstrating therapeutic equivalence between DDN and SDN because this trial was not designed as an equivalence or non-inferiority study and no equivalence margin was prespecified. Rather, no statistically significant between-group differences were detected for most outcomes in this sample, while a nominal between-group difference was observed for active RMS at Week 6. These findings provide a basis for reconsidering, rather than rejecting, strictly depth-dependent explanations of dry needling effects.

The clinical findings raise the hypothesis that direct intramuscular disruption may not be the sole pathway through which dry needling-related clinical changes occur. The SDN protocol was intended to remain superficial; however, actual insertion depth was not instrumentally verified. Moreover, the lack of sham or untreated control prevents attribution of the observed within-group improvements specifically to needling. Peripheral and central neurophysiological mechanisms described in the prior literature [8,16,45] should therefore be considered plausible explanatory hypotheses rather than mechanisms demonstrated by the present study.

Proposed mechanisms in the literature include segmental nociceptive modulation, activation of Aδ and C afferents, and engagement of descending inhibitory systems [45]. These mechanisms were not directly measured in the present trial. Future mechanistic studies incorporating appropriate control groups and direct neurophysiological measurements are required to determine whether such pathways account for clinical responses to superficial or deep dry needling.

### 4.2. Strengths and Limitations

A major strength of the current study is its randomized, two-arm parallel design, with participants and outcome assessors blinded to allocation. The study directly compared DDN and SDN using objective physiological outcomes, including ultrasonography and sEMG, in addition to patient-reported pain and disability. Outcomes were collected at baseline, Week 2 post-treatment, and Week 6, providing a 4-week treatment-free follow-up period after completion of the intervention.

Several limitations should be acknowledged. The treating therapist could not be blinded to the intervention delivered, creating a potential risk of performance bias. To reduce procedural variation, both groups followed the same treatment schedule, positioning, MTrP localization procedure, fast-in/fast-out approach, and prescribed 3–5 needle movements; nevertheless, therapist-related expectancy or interaction effects cannot be completely excluded. The absence of a sham-needling or untreated control group limits causal inference because within-group improvements may partly reflect nonspecific treatment effects, participant expectations, natural symptom fluctuation, or regression to the mean. MTrP identification relied on clinical palpation and therefore remained partly subjective. The SDN protocol required superficial insertion, but actual insertion depth was not instrumentally verified. The available records also did not document a prespecified number of repeated elastography acquisitions, a numerical post-preparation skin-impedance threshold, or exact needling duration in seconds. The study was not designed as an equivalence or non-inferiority trial, and multiple physiological outcomes were examined without multiplicity adjustment. The sample-size calculation targeted a moderate group-by-time effect (f = 0.25), so smaller between-group differences cannot be definitively excluded. Finally, the findings are specific to upper-trapezius MTrPs in people with non-specific neck pain and may not generalize to MTrPs in other muscles or other neck-pain subgroups. The study documentation also did not specify a standardized diagnostic-imaging pathway for eligibility, so unrecognized structural contributors cannot be definitively excluded.

## 5. Conclusions

Both DDN and SDN were associated with reductions in pain and disability, with no statistically significant between-group differences detected for these clinical outcomes in this sample. Most ultrasonographic and sEMG comparisons were also non-significant, although a nominally significant between-group difference in active RMS was observed at Week 6. Because the trial was not designed for equivalence or non-inferiority, these results do not establish therapeutic equivalence or demonstrate that deep needle penetration is unnecessary. The absence of consistent ultrasonographic changes may reflect either a true lack of measurable structural or vascular modification or limited sensitivity of strain elastography, B-mode imaging, and Doppler methods to subtle localized changes. Central neurophysiological mechanisms were not directly measured; the findings are therefore consistent with, but do not prove, contributions from peripheral or central modulation. The study was powered for a moderate between-group effect, and smaller differences between DDN and SDN cannot be ruled out. Further adequately powered controlled mechanistic studies are warranted.

## Figures and Tables

**Table 1:** Baseline demographic characteristics of study participants.

Characteristic	DDN (n = 22)	SDN (n = 22)	*p*-Value
**Demographics**			
Age (years)	42.02 ± 11.51	43.42 ± 11.18	0.684 *
Gender			1.000 ^#^
Male	12 (54.5%)	11 (50.0%)	
Female	10 (45.5%)	11 (50.0%)	
BMI (kg/m^2^)	27.66 ± 3.95	27.93 ± 4.52	0.953 *
Handedness			0.664 ^$^
Right	20 (90.9%)	18 (81.8%)	
Left	2 (9.1%)	4 (18.2%)	
**Clinical Characteristics**			
Neck pain duration (months)	39.52 ± 44.05	56.20 ± 92.47	0.715 ^@^
Affected side			0.763 ^#^
Right	11 (50.0%)	13 (59.1%)	
Left	11 (50.0%)	9 (40.9%)	
Smoking status			0.547 ^$^
Never	15 (68.2%)	17 (77.3%)	
Current smoker	5 (22.7%)	5 (22.7%)	
Former/Occasional	2 (9.1%)	0 (0.0%)	
Physical activity			0.440 ^#^
Inactive	10 (50.0%)	10 (45.5%)	
Insufficiently active	6 (30.0%)	4 (18.2%)	
Active	4 (20.0%)	8 (36.4%)	
**Comorbidities**			
None	14 (63.6%)	19 (86.4%)	0.344 ^$^
HTN	5 (22.7%)	2 (9.1%)	
DM	1 (4.5%)	1 (4.5%)	
G6PD	2 (9.1%)	0 (0.0%)	
Sickle cell anemia	0 (0.0%)	1 (4.5%)	
Asthma	1 (4.5%)	0 (0.0%)	
GERD	1 (4.5%)	0 (0.0%)	

Note. Continuous data are given as mean ± standard deviation (SD) and categorical variables as frequency, *n* (%). Abbreviations: BMI, body mass index; DDN, deep dry needling; DM, diabetic mellitus; GERD, gastroesophageal reflux disease; G6PD, glucose-6-phosphate dehydrogenase deficiency; HTN, hypertension; SDN, superficial dry needling. Statistical tests: ^#^, chi-square test; ^$^, Fisher’s exact test; *, independent-samples *t* test; ^@^, Mann–Whitney U test. For physical activity, percentages were calculated using available observations (DDN n = 20; SDN n = 22).

**Table 2:** Baseline, 2 weeks post-treatment and 6-week follow-up mean ± standard deviation (SD) values for main and secondary outcome measures.

Outcome	Group	Baseline	2 Weeks	6 Weeks
**Ultrasound Parameters**				
Muscle Stiffness (SR)	DDN	1.15 ± 0.20	1.15 ± 0.15	1.16 ± 0.19
	SDN	1.11 ± 0.20	1.04 ± 0.21	1.15 ± 0.31
Thickness (mm)	DDN	10.23 ± 2.93	10.88 ± 2.50	10.41 ± 1.61
	SDN	10.24 ± 1.21	10.30 ± 1.20	10.39 ± 1.57
BF-PSV (cm/s)	DDN	7.35 ± 2.93	7.32 ± 2.29	7.87 ± 1.80
	SDN	7.47 ± 2.97	8.60 ± 3.25	7.65 ± 2.96
BF-EDV (cm/s)	DDN	0.80 ± 0.62	0.70 ± 0.35	0.82 ± 0.51
	SDN	0.87 ± 0.58	0.80 ± 0.31	0.72 ± 0.44
BF-RI	DDN	0.89 ± 0.06	0.87 ± 0.12	0.89 ± 0.05
	SDN	0.88 ± 0.07	0.88 ± 0.11	0.90 ± 0.05
**sEMG Parameters**				
MVIC (normalized value)	DDN	0.48 ± 0.24	0.47 ± 0.19	0.63 ± 0.21
	SDN	0.42 ± 0.13	0.50 ± 0.13	0.55 ± 0.21
Rest RMS (μV)	DDN	4.28 ± 1.76	4.29 ± 1.86	4.55 ± 1.69
	SDN	4.51 ± 2.18	5.14 ± 1.86	5.29 ± 1.78
Active RMS (μV)	DDN	4.39 ± 2.11	3.92 ± 1.66	3.98 ± 1.32
	SDN	4.19 ± 1.97	3.18 ± 1.60	2.88 ± 1.69
Rest MDF (Hz)	DDN	65.41 ± 25.07	72.91 ± 26.00	75.45 ± 35.71
	SDN	68.94 ± 23.66	70.69 ± 27.47	69.05 ± 20.30
Active MDF (Hz)	DDN	83.36 ± 20.71	82.31 ± 12.79	81.65 ± 21.41
	SDN	77.50 ± 14.62	83.25 ± 15.43	79.57 ± 13.63
**Pain** (VAS [cm])	DDN	6.74 ± 2.27	3.08 ± 2.43	3.40 ± 2.76
	SDN	6.28 ± 2.10	3.24 ± 2.18	2.95 ± 2.15
**Disability** (NDI […/50])	DDN	16.68 ± 6.22	12.00 ± 7.67	12.89 ± 9.11
	SDN	15.59 ± 6.01	11.35 ± 6.18	9.72 ± 5.45

Note. Values are presented as Mean ± Standard Deviation. The randomized sample was DDN n = 22 and SDN n = 22. Available observed measurements were DDN/SDN 22/22 at baseline, 19/20 at Week 2, and 19/18 at Week 6; these observed counts do not alter the randomized group sizes. MVIC values are reported on the normalized 0–1 scale and are not percentages. BF = Blood Flow; DDN = Deep Dry Needling; EDV = End Diastolic Velocity; MDF = Median Frequency; MVIC = Maximum Voluntary Isometric Contraction; NDI = Neck Disability Index; PSV = Peak Systolic Velocity; RI = Resistive Index; RMS = Root Mean Square; SDN = Superficial Dry Needling; VAS = Visual Analogue Scale.

**Table 3:** Comparison of primary outcomes between both groups.

Variable	Group	2 Weeks Post-Treatment			6 Weeks Follow-Up		
		Change from Baseline Mean (95% CI)	Between-Group Difference (95% CI)	*p*-Value	Change from Baseline Mean (95% CI)	Between-Group Difference (95% CI)	*p*-Value
**Ultrasound Parameters**							
Muscle Stiffness (SR)	DDN	0.03 (−0.06, 0.11)	0.11 (−0.01, 0.24)	0.072	0.03 (−0.09, 0.15)	0.01 (−0.16, 0.18)	0.927
	SDN	−0.09 (−0.17, −0.001)			0.02 (−0.10, 0.15)		
Muscle Thickness (mm)	DDN	0.47 (−0.63, 1.56)	0.47 (−0.62, 1.56)	0.869	−0.02 (−0.64, 0.67)	−0.05 (−0.99, 0.88)	0.908
	SDN	0.002 (−0.76, 0.77)			0.07 (−0.60, 0.74)		
BF-PSV (cm/s)	DDN	0.09 (−1.20, 1.37)	−1.13 (−2.93, 0.68)	0.213	0.57 (−0.58, 1.72)	0.22 (−1.43, 1.87)	0.790
	SDN	1.21 (−0.04, 2.47)			0.35 (−0.83, 1.53)		
BF-EDV (cm/s)	DDN	−0.11 (−0.25, 0.02)	−0.08 (−0.27, 0.12)	0.426	−0.01 (−0.23, 0.22)	0.10 (−0.23, 0.42)	0.557
	SDN	−0.04 (−0.17, 0.10)			−0.10 (−0.33, 0.13)		
BF-RI	DDN	−0.02 (−0.07, 0.04)	0.01 (−0.01, 0.06)	0.810	0.003 (−0.02, 0.03)	−0.01 (−0.05, 0.02)	0.429
	SDN	−0.01 (−0.06, 0.04)			0.02 (−0.01, 0.04)		
**sEMG Parameters**							
MVIC (normalized value)	DDN	0.001 (−0.07, 0.08)	−0.05 (−0.16, 0.05)	0.321	0.15 (0.06, 0.25)	0.05 (−0.09, 0.19)	0.481
	SDN	0.05 (−0.02, 0.13)			0.10 (0.01, 0.20)		
Rest RMS (μV)	DDN	−0.17 (−1.04, 0.69)	−0.81 (−2.02, 0.40)	0.185	0.07 (−0.76, 0.89)	−0.69 (−1.87, 0.49)	0.241
	SDN	0.64 (−0.21, 1.48)			0.76 (−0.08, 1.61)		
Rest MDF (Hz)	DDN	7.67 (−3.18, 18.5)	6.06 (−9.15, 21.3)	0.808	8.86 (−4.85, 22.58)	7.98 (−11.8, 27.8)	0.819
	SDN	1.60 (−8.96, 12.16)			0.89 (−13.2, 15.0)		
Active RMS (μV)	DDN	−0.45 (−1.17, 0.28)	0.52 (−0.50, 1.54)	0.305	−0.28 (−1.0, 0.44)	1.09 (0.06, 2.13)	0.039 *
	SDN	−0.97 (−1.67, −0.26)			−1.38 (−2.11, −0.64)		
Active MDF (Hz)	DDN	0.87 (−4.3, 6.04)	−3.35 (−10.6, 3.9)	0.356	0.08 (−6.92, 7.08)	0.01 (−9.98, 10.0)	0.998
	SDN	4.22 (−0.84, 9.28)			0.06 (−7.04, 7.17)		

Note. Data is given as estimated marginal means (95% confidence intervals [CIs]), adjusted for the matching baseline covariate. The difference between groups was computed as DDN minus SDN, with SDN as the reference. Abbreviations DDN, deep dry needling; SDN, superficial dry needling; CI, confidence interval; BF, blood flow; PSV, peak systolic velocity; EDV, end-diastolic velocity; RI, resistive index; MVIC, maximal voluntary isometric contraction; RMS, root mean square; MDF, median frequency. * Statistically significant Active RMS was interpreted on the original change-from-baseline scale in the final SPSS output. No formal multiplicity adjustment was applied; the *p* = 0.039 active-RMS contrast is therefore considered nominal/exploratory.

**Table 4:** Comparison of secondary outcomes between both groups.

Variable	Group	2 Weeks Post-Treatment			6 Weeks Follow-Up		
		Change from Baseline Mean (95% CI)	Between-Group Difference (95% CI)	*p*-Value	Change from Baseline Mean (95% CI)	Between-Group Difference (95% CI)	*p*-Value
**Pain** (VAS [cm])	DDN	−3.35 (−4.30, −2.40)	−0.27 (−1.60, 1.06)	0.685	−3.04 (−4.04, −2.04)	0.26 (−1.17, 1.69)	0.714
	SDN	−3.08 (−4.01, −2.16)			−3.30 (−4.32, −2.28)		
**Disability** (NDI […/50])	DDN	−5.4 (−7.9, −2.9)	−0.43 (−3.9, 3.1)	0.805	−4.5 (−7.3, −1.8)	1.93 (−2.0, 5.9)	0.328
	SDN	−5.0 (−7.4, −2.5)			−6.5 (−9.3, −3.6)		

Note. “Values represent estimated marginal means” (95% CI) adjusted for the respective baseline covariate. Between-group differences represent DDN minus SDN. DDN = Deep Dry Needling; SDN = Superficial Dry Needling (reference group); CI = confidence interval; NDI = Neck Disability Index; VAS = Visual Analogue Scale.

**Table 5:** Comparison of minor adverse events between both groups.

Adverse Event	DDN (n = 22), n (%)	SDN (n = 22), n (%)	*p*-Value
Bleeding	8 (36.4%)	0 (0.0%)	0.004 *
Bruising	4 (18.2%)	0 (0.0%)	**0.108**
Pain During Needling	17 (77.3%)	9 (40.9%)	0.031 *
Pain After Needling	14 (63.6%)	6 (27.3%)	0.033 *
Temporary Aggravated Symptoms	4 (18.2%)	2 (9.1%)	**0.664**
Drowsiness	2 (9.1%)	0 (0.0%)	**0.488**
Fatigue	4 (18.2%)	1 (4.5%)	**0.345**
Headache	1 (4.5%)	0 (0.0%)	**1.000**
Nausea	0 (0.0%)	0 (0.0%)	**1.000**
Sweating	1 (4.5%)	0 (0.0%)	**1.000**

Note. Adverse events were monitored across four treatment sessions for each participant. Data are presented as the number and percentage of participants who experienced each adverse event at least once during the treatment period, n (%). Between-group comparisons were performed using the chi-square test or Fisher’s exact test, as appropriate, particularly when expected cell frequencies were small. Abbreviations: DDN, deep dry needling; SDN, superficial dry needling. Percentages represent participants experiencing each event at least once across the four sessions, not per-session event rates. * Statistically significant between-group difference.

## Data Availability

The data that supports the findings of this study are openly available in the “Figshare repository at 10.6084/m9.figshare.33210777”.

## Supplementary Materials

The following supporting information can be downloaded at https://www.mdpi.com/article/10.3390/bios16100583/s1, Figure S1, Pain intensity (VAS) in the deep dry needling (DDN) and superficial dry needling (SDN) groups at baseline, 2 weeks post-treatment, and 6 weeks follow-up. Bars represent mean values and error bars represent standard deviations. Figure S2, Neck Disability Index (NDI) in the deep dry needling (DDN) and superficial dry needling (SDN) groups at baseline, 2 weeks post-treatment, and 6 weeks follow-up. Bars represent mean values and error bars represent standard deviations.
