# Supplementary material for: Ultrasonographic and Electromyographic Evaluation of the Effects of Deep Versus Superficial Dry Needling on Myofascial Trigger Points in Patients with Neck Pain: A Triple-Blind Randomized Clinical Trial
Source: Biosensors (Basel). 2026 Oct 18;16(10):583. doi: 10.3390/bios16100583 (PMC13702262; doi:10.3390/bios16100583)
Supplement: Supplementary file 1 [file biosensors-16-00583-s001.zip › biosensors-4549111-supplementary.pdf]

Supplementary Materials

Ultrasonographic and electromyographic evaluation of the effects of deep versus superficial dry needling on myofas-cial trigger points in patients with neck pain: A triple-blind randomized clinical trial

Anas Mohammed Alhakami [1,*], Diyaa Abdul Rauf Algazwi [2], Majdi Habib Al-Awjami [3], Walaa Hamdy Elsayed [1] and Ali Muteb Alshami [1]

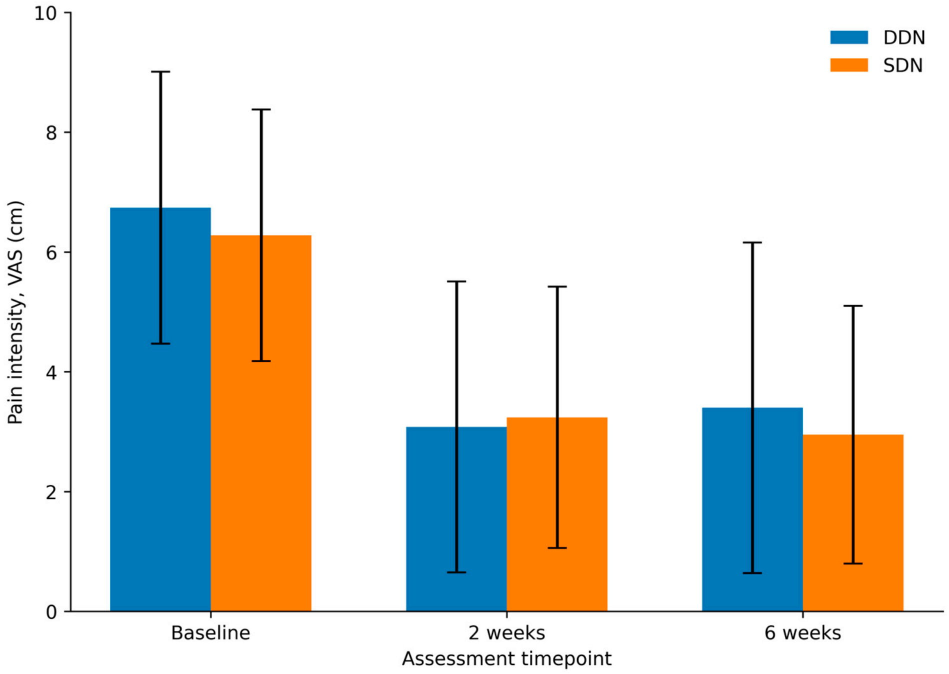

Figure S1. Pain intensity (VAS) in the deep dry needling (DDN) and superficial dry needling (SDN) groups at baseline, 2 weeks post-treatment, and 6 weeks follow-up. Bars represent mean values and error bars represent standard deviations.

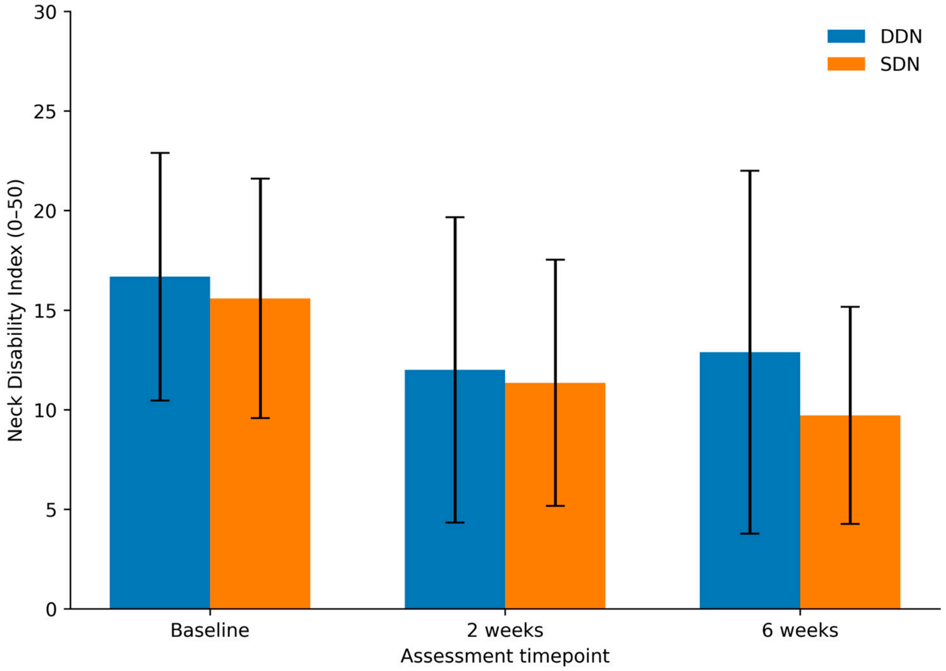

Figure S2. Neck Disability Index (NDI) in the deep dry needling (DDN) and superficial dry needling (SDN) groups at baseline, 2 weeks post-treatment, and 6 weeks follow-up. Bars represent mean values and error bars represent standard deviations.
